# A Meta-Analysis of Patient-Reported Outcomes of Sacituzumab Govitecan Versus Treatment of Physician’s Choice in Previously Treated HR+/HER− mBC Using Two Phase 3 (TROPiCS-02 and EVER-132-002) Trials

**DOI:** 10.3390/cancers17111885

**Published:** 2025-06-04

**Authors:** Hope S. Rugo, Binghe Xu, Anandaroop Dasgupta, Ankita Kaushik, Wendy Verret, Barinder Singh

**Affiliations:** 1 Helen Diller Family Comprehensive Cancer Center, University of California, San Francisco, CA 94143, USA; 2 Department of Medical Oncology, Cancer Hospital Chinese Academy of Medical Sciences and Peking Union Medical College, Beijing 100021, China; 3 Gilead Sciences, Inc., Foster City, CA 94404, USA; anandaroop.dasgupta1@gilead.com (A.D.);; 4 Pharmacoevidence, London SE1 0AS, UK

**Keywords:** HR+/HER2 mBC, metastatic breast cancer, sacituzumab govitecan, TROPiCS-02, EVER-132-002, quality of life, patient-reported outcomes, EORTC QLQ-C30, EQ-5D-5L VAS

## Abstract

Background: The patient-reported outcomes (PROs) of sacituzumab govitecan (SG) were compared with chemotherapy using two phase 3 trials (TROPiCS-02, EVER-132-002) involving patients with HR+/HER2− locally recurrent inoperable or metastatic breast cancer. Methods: A meta-analysis was performed to compare change from baseline (CFB) scores and time-to-deterioration (TTD) between SG and chemotherapy using EORTC QLQ-C30 and EQ-5D-5L VAS in the overall, prior CDK4/6i-treated, and fast-progressor populations. Results of CFB and TTD analyses were summarized using hazard ratio (HR) and mean difference measures. Results: Statistically significant improvement (*p* < 0.05) in CFB scores was observed with SG over chemotherapy in five EORTC QLQ-C30 domains: physical (mean difference: 2.64), role functioning (mean difference: 2.70), fatigue (mean difference: −2.51), pain (mean difference: −3.25) and dyspnea (mean difference: −3.27), and EQ-5D-5L VAS (mean difference: 1.58). In the overall population, longer TTD (*p* < 0.05) was observed with SG versus chemotherapy on six domains of EORTC QLQ-C30: GHS/QoL (HR: 0.76), physical (HR: 0.72), emotional functioning (HR: 0.73), fatigue (HR: 0.80), pain (HR: 0.82), and dyspnea (HR: 0.71). Results from EORTC QLQ-C30 domains were mostly consistent among the overall, prior CDK4/6i treated and fast-progressor populations. SG demonstrated longer TTD (*p* < 0.05) over chemotherapy for EQ-5D-5L-VAS across all studied populations (HR range: 0.63–0.69). PROs significantly worsened with SG in the domains of diarrhea and nausea and vomiting (commonly reported adverse events of SG, manageable by following established guidelines). Conclusions: SG significantly improved PROs versus chemotherapy for several subdomains of EORTC QLQ-C30 and EQ-5D-5L-VAS. The consistency of these results in the overall population and subgroups supports the generalizability of the meta-analytic evidence and reinforces the PRO benefits associated with SG versus chemotherapy.

## Simple Summary

Breast cancer is the most common type of cancer among women, with HR+/HER2− disease representing ~70% of all breast cancers. There is a lack of documented evidence showing humanistic outcomes (changes in patient quality of life/functional status) associated with treatment in an HR+/HER2− population, which can be generalized to the global level. This study included two clinical studies, TROPiCS-02 and EVER-132-002, involving patients with HR+/HER2− locally recurrent inoperable or metastatic breast cancer who were treated with sacituzumab govitecan or chemotherapy of physician’s choice. We compared the patient-reported outcomes of sacituzumab govitecan versus chemotherapy in the overall population, the CDK4/6 inhibitor pre-treated population, and in patients with fast-progressing disease. Sacituzumab govitecan was found to demonstrate patient-reported outcome benefits compared with chemotherapy in different patient subgroups. These findings support the use of sacituzumab govitecan as a standard of care for pre-treated HR+/HER2− metastatic breast cancer regardless of previous CDK4/6 inhibitor treatment.

## 1. Background

Breast cancer is the most common cancer among females and the second leading cause of death in the United States (US) [1,2]. Hormone receptor-positive/human epidermal growth factor receptor 2 negative metastatic breast cancer (HR+/HER2− mBC) is the most common subtype of breast cancer, accounting for approximately 70% of breast cancers [3]. Endocrine therapy serves as a cornerstone treatment approach for systemic treatment in HR+/HER2− breast cancer, followed by aromatase inhibitors, selective estrogen receptor degraders, CDK4/6 inhibitors (CDK4/6i), and selective estrogen receptor modulators, which have also demonstrated clinical benefits [4]. However, in patients who become resistant to endocrine treatment, chemotherapy becomes the primary initial treatment choice; yet, chemotherapy has modest efficacy and is associated with poor quality of life (QoL) and toxicity in later lines [5].

Antibody–drug conjugates are emerging as a new therapeutic choice in treating breast cancer [6]. Sacituzumab govitecan (SG) is an antibody–drug conjugate with approved indications in the metastatic HR+/HER− and triple-negative breast cancer populations [7,8].

Two randomized, open-label, and multicentered phase 3 clinical trials, TROPiCS-02 (NCT03901339) and EVER-132-002 (NCT04639986), were conducted to evaluate the patient-reported outcomes (PROs) of SG versus treatment of physician’s choice (TPC) in patients with HR+/HER− locally recurrent inoperable or mBC, after failure of at least two but no more than four prior chemotherapy regimens for metastatic disease [7,9]. In the TROPiCS-02 trial, the SG arm had a lower proportion of patients with a clinically meaningful worsening in primary domains of European Organization for Research and Treatment of Cancer Quality of Life Questionnaire Version 3 (EORTC QLQ-C30) including global health status/QoL (GHS/QoL), physical functioning, role functioning, and fatigue, secondary domains of social functioning and insomnia and EuroQol 5 Dimensions Visual Analog Scale (EQ-5D-5L VAS) compared with TPC. The time to first clinically meaningful worsening or death significantly favored the SG arm for GHS/QoL (hazard ratio (HR) [95% CI] 0.75 [0.61 to 0.92]; *p* = 0.006), physical functioning (0.79 [0.64 to 0.97]; *p* = 0.022), and fatigue (0.73 [0.60 to 0.89]; *p* = 0.002). Moreover, the overall least square mean change from baseline (CFB) was significantly better for SG for physical functioning and dyspnea [5]. In the EVER-132-002 trial, PROs were favorable for SG over TPC, consistent with the TROPiCS-02 study [7,10].

While improvement in PROs/QoL with SG has been demonstrated in multiple clinical studies, there is an unmet need to understand the generalizability of these data across the US, Europe, and Asia. We report a post hoc analysis of the two trials TROPiCS-02 (conducted in the US and Europe) and EVER-132-002 (conducted in Asia) to compare the PROs for SG versus TPC. This multiregional study will provide generalizability of results associated with PROs among SG-treated patients in the HR+/HER− mBC population. Evidence generated from this meta-analysis will help elucidate the PROs in diverse populations and inform clinical practice and guidelines.

## 2. Materials and Methods

The current meta-analysis was conducted according to the Preferred Reporting Items for Systematic Reviews and Meta-Analyses of Individual Participant Data (PRISMA-IPD) guidelines [11].

### 2.1. Selection Criteria

Two randomized controlled phase 3 trials, TROPiCS-02 and EVER-132-002, which assessed SG versus TPC in patients with HR+/HER− mBC who had previously received endocrine therapy, taxane, and at least two systemic therapies in the advanced setting were included in the current study. Primary data and the protocols for these studies have been previously published [7,12]. In order to match the TROPiCS-02 population, the subgroup of prior CDK4/6i-treated patients from EVER-132-002 was considered for the feasibility of the meta-analysis. The detailed information on these trials has been previously published [3,7,9]. The eligibility criteria for SG versus TPC meta-analyses are summarized in Table 1. The included patients were required to have a baseline score of (i) ≥10 for functional and GHS/QoL domains, (ii) ≤90 for symptom scales of the EORTC QLQ-C30 [13,14,15], and (iii) ≥15 for EQ-5D-5L Visual Analog Scale (VAS) [16].

### 2.2. Risk-of-Bias Assessment

The eligible randomized controlled trials were assessed for bias using the Cochrane Collaboration Risk of Bias Tool (RoB 2.0) according to the Cochrane Handbook for Systematic Reviews of Interventions [17].

### 2.3. Feasibility Assessment

Meta-analysis relies on the transitivity assumption, which necessitates that the different sets of studies of an indirect comparison are, on average, similar in all key factors that could influence the relative treatment effects [18,19]. Hence, a feasibility assessment of the meta-analytic framework was performed by assessing the availability of the evidence and heterogeneity of prognostic factors at trial and patient level along with approaches for meta-analyzing the effect sizes of SG versus TPC from the TROPiCS-02 and EVER-132-002 trials. The feasibility process involved several steps. Initially, heterogeneous variables were identified by regression methods and by seeking clinician feedback. This was followed by an assessment of statistical heterogeneity, which was assessed using the I^2^ statistic [17] and selection of a meta-analytic model based on outcome type and heterogeneity assessment [20,21]. In chi-square analysis, a *p*-value < 0.05 indicates statistical significance, while a *p*-value > 0.05 indicates no statistical significance.

According to the PRISMA-IPD guidelines [11], the individual patient data (IPD), collected by the sponsor during the studies of interest, were assessed for integrity, consistency, baseline imbalances, and missing values. By using the IPD, various subgroups were evaluated based on effect modifiers, allowing for a determination of whether certain individuals benefit more than others from the intervention.

### 2.4. PRO Assessments

In the TROPiCS-02 trial, time to deterioration (TTD) was analyzed for the EORTC QLQ-C30 GHS/QoL, pain, and fatigue scales, and was defined as the time from randomization to the first date a patient had a ≥10-point deterioration from baseline or death from any cause, whichever occurred first. Moreover, patients needed to have a baseline GHS/QoL score ≥ 10, baseline pain score ≤ 90, and baseline fatigue score ≤ 90, respectively, to be included. The PROs were assessed in all patients at baseline (within 3 days of the first study treatment), Day 1 of every cycle except Cycle 1 (every 3 weeks for TPC if given weekly), and the final study visit (prior to telling patients that they are being withdrawn from the study). During the follow-up period, PROs were measured every 60 days [15,22]. The Kaplan–Meier method was used to estimate TTD, with treatment groups compared using a stratified log-rank test. HRs and 95% CIs were obtained from a stratified Cox proportional-hazards model. Patients who never experienced clinically meaningful worsening were censored at the time of their last non-missing assessment. Death was considered as an event in the main analysis but censored at the time of the last non-missing PRO assessment in the sensitivity analysis. Death is treated as a censored event to ensure the analysis reflects only observed declines in health status as directly reported by the patient. The overall within-group least-square mean CFB and between-treatment difference in least-square mean change was assessed using linear mixed-effects models for repeated measures (MMRM) with random intercept and time effects [23]. Similarly, in the EVER-132-002 trial, observed scores and changes from baseline for each EORTC QLQ-C30 scale were summarized using descriptive statistics by analysis visit for the PRO-evaluable population. The PRO-evaluable and EQ-5D-5L evaluable population were defined as all the randomized patients who had an evaluable assessment of health-related quality of life or EQ-5D-5L at baseline and at least one evaluable assessment at post-baseline visits. TTD for GHS/QoL, pain, fatigue, and CFB in physical and role functioning were defined and analyzed in the same way as in the TROPiCS-02 trial, including patients with baseline scores that met specific criteria [13].

In the current study, PROs were assessed using the EORTC QLQ-C30 and EQ-5D-5L VAS instruments in the overall, prior CDK4/6i-treated, and fast-progressors population (duration of prior CDK4/6i treatment ≤ 12 months). The EORTC QLQ-C30 questionnaire is a 30-item cancer-specific questionnaire consisting of five functional scales (physical, role, cognitive, emotional, and social functioning), three symptom scales (fatigue, pain, nausea and vomiting), six single items (dyspnea, appetite loss, sleep disturbance, constipation, diarrhea, and financial difficulties) and one two-item GHS/QoL domain. For the current analysis, a summary score for the EORTC QLQ-C30 was also calculated as the mean score for 13 of the 15 domains, excluding the GHS/QoL and financial difficulties. Each domain, including the summary score, had a score range of 0 to 100 [24]. The EQ-5D measure includes an EQ-5D-5L scale and a VAS. The EQ-5D-5L assesses five dimensions of health including mobility, self-care, usual activities, pain/discomfort, and anxiety/depression [25,26]. The scheduled visits were defined for the PRO-evaluable population and EQ-5D-5L evaluable population. An evaluable assessment at a given visit was defined as at least one of the fifteen domains/scales of the EORTC QLQ-C30 and at least one of the five dimensions of the EQ-5D-5L VAS score being non-missing at that scheduled visit. The two-stage method was the primary analysis method for the CFB due to differences in the timing of unscheduled visits between TROPiCS-02 and EVER-132-002 trials. These unscheduled visits often happen due to patient safety concerns or side effects, causing assessments to be performed at different times and frequencies in each trial. This resulted in inconsistent collection and reporting of CFB data, leading to a lack of uniformity. The one-stage approach when not including death in the analysis (time of the last non-missing PRO assessment before death in the analysis) was the primary analysis method (base case) for TTD. Furthermore, the sensitivity analysis for CFB was performed using the one-stage method, and for TTD, the sensitivity analysis was performed using the one-stage method after including death as an event.

### 2.5. Statistical Analysis

The EORTC QLQ-C30 and EQ-5D-5L VAS CFB meta-analysis was performed using MMRM and mean difference methods. MMRM was performed to estimate the treatment effect on CFB in the physical function and role function scale using a PRO-evaluable population. The MMRM model included baseline score, treatment, analysis visit, stratification factors, and treatment by analysis visit interaction as fixed effects (FEs), with the intercept and analysis visit as random effects (REs) along with all QLQ-C30 analysis visits up to (but not including) the first analysis visit at which the sample size in one of the treatment arms was below 25 patients and assessed while patients remained on treatment (i.e., excluding end of treatment visit). Least square mean difference (LSMD) was used to measure the adjusted difference between treatment groups, considering factors like baseline scores and visits, to provide a more accurate treatment effect. The mean difference method applied descriptive statistics to summarize the observed scores at each analysis visit and the CFB scores at each post-baseline visit for each domain of the EORTC QLQ-C30 and EQ-5D-5L VAS. This analysis was conducted by treatment group within the PRO-evaluable population and reflected the unadjusted difference in average scores between groups at specific visits, offering a simple comparison. For functional domains, a positive LSMD/mean difference indicated improved function, while a negative value reflected a decline in function, whereas, for symptom domains, a positive value suggested worsening symptoms and a negative value indicated symptom improvement. The HR and 95% CI for TTD were estimated using a stratified Cox proportional hazards regression analysis. A clinically meaningful deterioration was defined as at least 10 points of worsening from baseline for EORTC QLQ-C30 and at least 15 points of worsening from baseline for EQ-5D-5L VAS.

The meta-analysis was conducted through IPD using both one-stage and two-stage IPD meta-regression models. In the one-stage approach, data from multiple clinical trials are pooled and evaluated simultaneously, offering the advantage of leveraging individual-level data across studies. However, to address potential sources of unobserved confounding, particularly those linked to variations in geographic regions and patient visit patterns between the TROPiCS-02 and EVER-132-002 trials, the two-stage method was selected for this analysis. In the two-stage IPD meta-analysis, individual-level data from each study are first analyzed separately. The aggregated results from these separate analyses are then combined in the second stage to generate an overall estimate [27,28]. This approach allows us to retain the integrity of trial-specific characteristics, ensuring that differences inherent to each trial are preserved before combining results. The choice of the two-stage method was essential given the distinct geographic and operational contexts of the two trials. TROPiCS-02 was conducted primarily in the US and Europe, while EVER-132-002 was conducted in Asian regions, with different health care settings and patient visit schedules. Direct pooling of data using a one-stage approach could obscure these contextual differences, introducing a risk of unobserved confounding. By applying a two-stage method, we minimize this risk, ensuring that regional and operational variations do not bias the overall results.

In a two-stage meta-analysis, choosing between FE and RE models is important because it affects the reliability and applicability of the results. The FE model assumes that one true effect size is the same across all studies, making it suitable when the trials are similar in terms of participants, treatments, and outcomes. In this model, each study provides a point estimate and variance in the first stage, and these are combined in the second stage to produce a single pooled effect size with narrower confidence intervals (CIs), indicating higher precision. On the other hand, the RE model recognizes that there may be different true effect sizes among studies due to variations in populations or methods, making it suitable when there is expected variability. Moreover, the RE model also calculates point estimates and variances in the first stage, but it combines these in the second stage by considering both within-study and between-study variances. This results in a pooled effect size with wider CIs, reflecting the uncertainty from heterogeneity. Therefore, while the FE model is more precise but less generalizable, the RE model allows for broader application of findings across different studies [26]. In cross-trial comparisons, the choice between FE and RE models depends on the degree of similarity between the trials. If the studies are expected to estimate a common effect, an FE model is considered appropriate. Conversely, if differences between trials are expected (e.g., in terms of population or intervention), an RE model would be more suitable to account for heterogeneity [17]. We addressed missing data using a complete case analysis, including only patients with complete information for all relevant covariates. This approach aligns with our goal of minimizing confounding effects and ensuring the reliability of our findings across trials.

## 3. Results

### 3.1. Study Characteristics and Risk-of-Bias Assessment

A low to moderate heterogeneity was reported across the included studies.

Overall, 874 patients were randomized in TROPiCS-02 (N = 543) and EVER-132-002 trials (N = 331). In TROPiCS-02, patients were recruited from the US, France, Spain, Germany, Belgium, Italy, Great Britain, the Netherlands, and Canada, whereas in the EVER-132-002 trial, participants were from the geographical regions of China, Republic of Korea, and Taiwan. Further, the PRO-evaluable population included 82.1% (n = 446) and 96.0% (n = 318) of patients in TROPiCS-02 and EVER-132-002 trials, respectively (Table 2). Prior CDK4/6i treatment status was a mandatory inclusion criterion for TROPiCS-02, whereas in EVER-132-002, only 161 (48.6%) patients with prior CDK4/6i treatment status were included. TROPiCS-02 trial recruited patients globally across Europe and North America and therefore contained a relatively smaller proportion of patients of Asian descent compared with the EVER-132-002 trial (2.9% versus 100%).

Across both TROPiCS-02 and EVER-132-002, mean age was comparable (56.4 years and 52.1 years, respectively), with a majority of the population < 65 years of age (74.2% and 88.5%), having an Eastern Cooperative Oncology Group performance status of 1 (55.6% and 77.6%), and receiving more than one prior line of chemotherapy in the metastatic setting (98.0% and 94.6%). Table 2 presents the characteristics of the pooled population, which was used in the meta-analysis. The baseline characteristics for both trials have been summarized in Supplementary Table S1.

Both studies were open-label, phase III randomized controlled trials with an overall low risk of bias across multiple domains including randomization process, deviations from intended interventions, missing outcome data, measurement of the outcome, and selection of the reported result.

### 3.2. Least Square Mean Changes from Baseline

**Figure 1:**
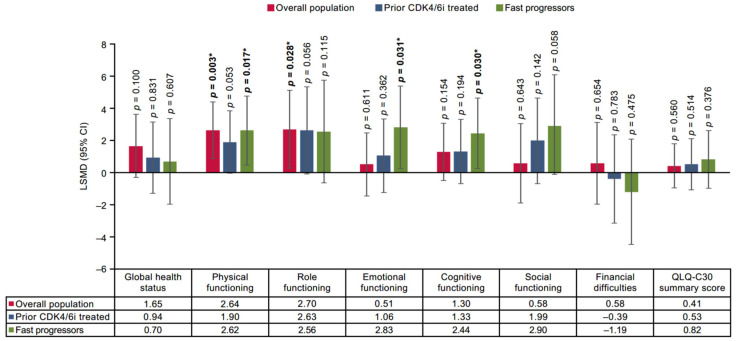
Treatment comparison of mean CFB in functional domains, GHS, summary scores, financial difficulties of EORTC QLQ-C30 of overall, prior CDK4/6i-treated, and fast-progressors population. * Indicate significant *p*-values (*p* < 0.05). Overall population: For EVER-132-002, the last time point considered was Week 19, and for TROPiCS-02, it was Cycle 11 Day 1. Prior CDK4/6i-treated population: For EVER-132-002, the last time point considered was Week 7, and for TROPiCS-02, it was Cycle 11 Day 1. Fast-progressors: For EVER-132-002, the last time point considered was Week 7, and for TROPiCS-02, it was Cycle 9 Day1. CDK4/6i = cyclin-dependent kinase 4/6 inhibitor; CFB = change from baseline; CI, confidence interval; EORTC QLQ-C30 = European Organization for Research and Treatment of Cancer Quality of Life Questionnaire version 3.0; GHS = global health status; LSMD = least square mean difference.

**Figure 2:**
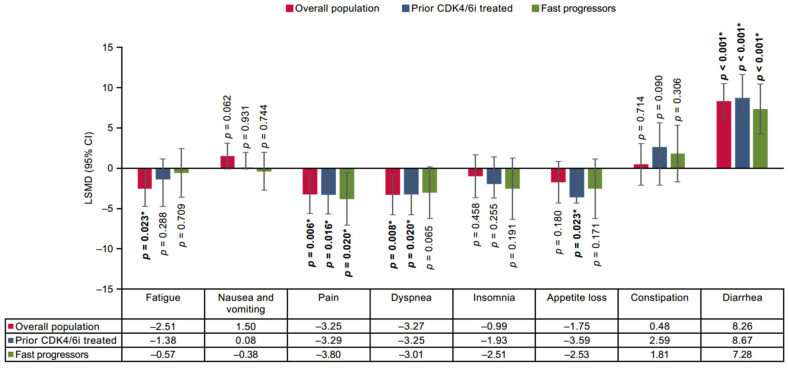
Treatment comparison of mean CFB in symptom domain scores of EORTC QLQ-C30 in overall, prior CDK4/6i-treated, and fast-progressors populations. * Indicate significant *p*-values (*p* < 0.05). Overall population: For EVER-132-002, the last time point considered was Week 19, and for TROPiCS-02, it was Cycle 11 Day 1. Prior CDK4/6i-treated population: For EVER-132-002, the last time point considered was Week 7, and for TROPiCS-02, it was Cycle 11 Day 1. Fast-progressors: For EVER-132-002, the last time point considered was Week 7, and for TROPiCS-02, it was Cycle 9 Day1. CDK4/6i = cyclin-dependent kinase 4/6 inhibitor; CFB = change from baseline; CI, confidence interval; EORTC QLQ-C30 = European Organization for Research and Treatment of Cancer Quality of Life Questionnaire Version 3.0; LSMD = least square mean difference.

The findings of the base case two-stage analysis, chosen to minimize the risk of bias, reported that in the overall population, SG demonstrated a statistically significant improvement versus TPC in two functional domains including physical functioning (mean difference: 2.64 [95% CI 0.88–4.41]; *p* = 0.003) and role functioning (2.70 [0.29 to 5.12]; *p* = 0.028) (Figure 1), and three symptom domains of EORTC QLQ-C30 including fatigue (−2.51 [–4.68 to −0.35]; *p* = 0.023), pain (–3.25 [–5.57 to −0.94]; *p* = 0.006), and dyspnea (–3.27 [–5.69 to −0.84]; *p* = 0.008) (Figure 2). The remaining domains did not exhibit statistically significant differences in improvement between SG and TPC.

**Figure 3:**
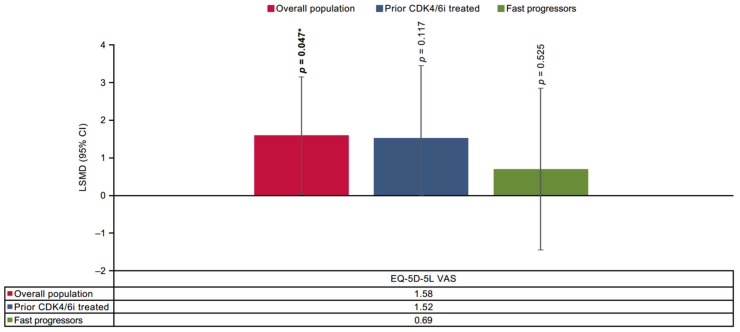
Treatment comparison of mean CFB EQ-5D-5L VAS in overall, prior CDK4/6i-treated and fast-progressors populations. * Indicate significant *p*-values (*p* < 0.05). CDK4/6i = cyclin-dependent kinase 4/6 inhibitor; CFB = change from baseline; CI = confidence interval; EQ-5D-5L VAS = EuroQol 5 Dimensions 5 Levels Visual Analog Scale; LSMD = Least square mean difference.

Furthermore, a statistically significant improvement with SG was reported in three symptom domains, including pain (–3.29 [–5.96 to −0.61]; *p* = 0.016), dyspnea (–3.25 [−5.99 to −0.51]; *p* = 0.020), appetite loss (–3.59 [–6.69 to −0.49]; *p* = 0.023) in the prior CDK4/6i-treated population. For the fast-progressors subgroup, statistically significant improvement with SG was reported in three functional domains and one symptom domain (physical functioning: 2.62 [0.47 to 4.77]; *p* = 0.017, emotional functioning: 2.83 [0.26 to 5.40]; *p* = 0.031, cognitive functioning: 2.44 [0.24 to 4.64]; *p* = 0.030, pain: −3.80 [–7.01 to −0.60]; *p* = 0.020) (Figure 1 and Figure 2). In all three populations, SG demonstrated non-significant improvement in several of the remaining functional and symptom domains, except diarrhea. Moreover, a statistically significant improvement in PROs with SG was also demonstrated on the EQ-5D-5L-VAS scale for the overall population compared with TPC (1.58 [0.02 to 3.14]; *p* = 0.047), while prior CDK4/6i and fast-progressors subgroups showed non-significant trends toward improvement in PROs with SG (Figure 3).

### 3.3. Mean Difference

**Figure 4:**
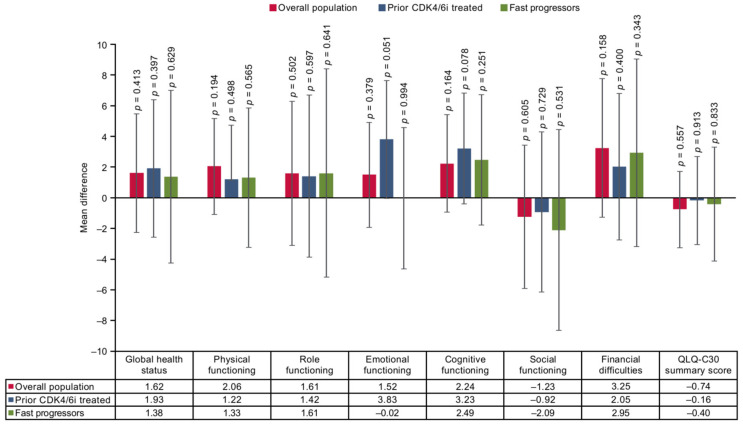
Treatment comparison of mean CFB in functional domains, GHS, summary scores, financial difficulties of EORTC QLQ-C30 of overall, prior CDK4/6i-treated and fast-progressors population at the end of treatment. CDK4/6i = cyclin-dependent kinase 4/6 inhibitor; CFB = change from baseline; EORTC QLQ-C30 = European Organization for Research and Treatment of Cancer Quality of Life Questionnaire Version 3.0.

**Figure 5:**
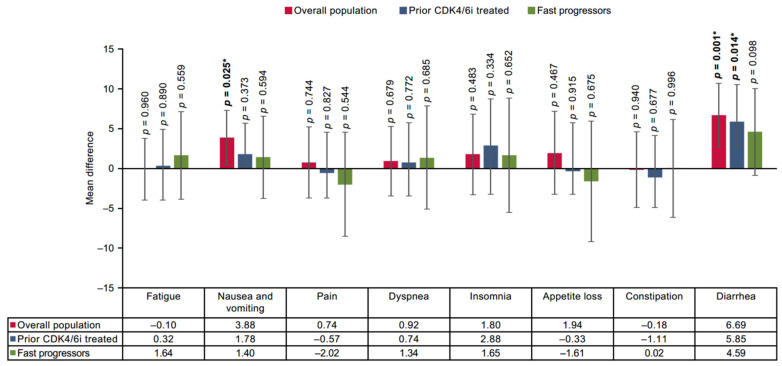
Treatment comparison of mean CFB in symptom domain scores of EORTC QLQ-C30 in overall, prior CDK4/6i-treated and fast-progressors population at the end of treatment. * Indicate significant *p*-values (*p* < 0.05). CDK4/6i = cyclin-dependent kinase 4/6 inhibitor; CFB = change from baseline; EORTC QLQ-C30 = European Organization for Research and Treatment of Cancer Quality of Life Questionnaire Version 3.0.

**Figure 6:**
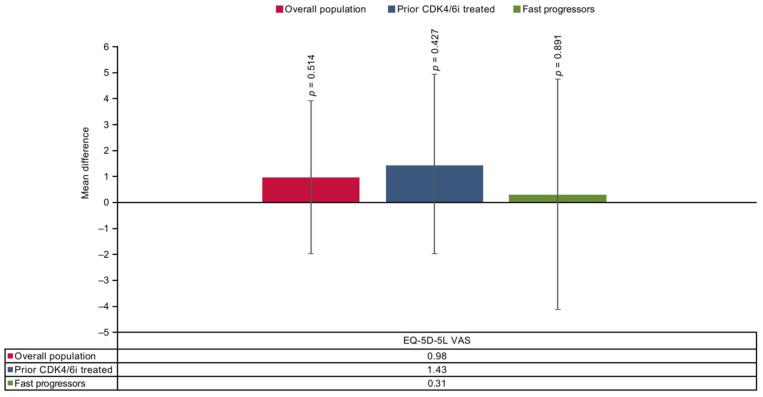
Treatment comparison of mean CFB EQ-5D-5L VAS in overall, prior CDK4/6i-treated and fast-progressors population at the end of treatment. CDK4/6i = cyclin-dependent kinase 4/6 inhibitor; CFB = change from baseline; EQ-5D-5L VAS = EuroQoL Five Dimensions Five Levels Visual Analog Scale.

The findings of the base case two-stage method reported no significant differences between SG and TPC arms on the EORTC QLQ-C30 scale at end of treatment except for diarrhea, nausea, and vomiting, which worsened significantly with SG in the overall population. In the prior CDK4/6i-treated population, SG showed a significant difference in CFB scores in the diarrhea symptom domain. However, for the fast-progressors subgroup, no significant differences were reported in any of the domains (Figure 4 and Figure 5). In all three populations, SG demonstrated non-significant improvement on the EQ-5D-5L VAS scale compared with TPC (Figure 6).

### 3.4. Time to Deterioration

The findings of the base case one-stage analysis after not including death as an event revealed that SG demonstrated a statistically significant increase in TTD compared with TPC for six of 15 domains of EORTC QLQ-C30, including GHS/QoL (HR:0.76 [95% CI 0.63 to 0.92]; *p* = 0.005), physical functioning (0.72 [0.59 to 0.88]; *p* = 0.001), emotional functioning (0.73 [0.58 to 0.91]; *p* = 0.006), fatigue (0.80 [0.67 to 0.95]; *p* = 0.011), pain (0.82 [0.67 to 0.99]; *p* = 0.042), and dyspnea (0.71 [0.57 to 0.88]; *p* = 0.002) measures in the overall population (Figure S1A). Similar findings were reported for the prior CDK4/6i-treated population (GHS/QoL: 0.69 [0.56 to 0.86]; *p* = 0.001, physical functioning: 0.74 [0.59 to 0.93]; *p* = 0.009, emotional functioning: 0.64 [0.49 to 0.83]; *p* = 0.001, fatigue: 0.77 [0.63, to 0.94]; *p* = 0.009, pain (0.77 [0.62 to 0.96]; *p* = 0.021, and dyspnea: 0.69 [0.54 to 0.88]; *p* = 0.003) (Figure S1B). For the remaining domains, a non-significant trend in TTD extension was reported for role functioning, cognitive functioning, social functioning, and insomnia, while for the two domains, diarrhea, nausea, and vomiting worsened with SG. Furthermore, subgroup results for fast-progressors revealed significant TTD findings for GHS/QoL (0.62 [0.47 to 0.82]; *p* = 0.001), physical functioning (0.69 [0.51 to 0.92]; *p* = 0.010), emotional functioning (0.53 [0.38 to 0.74]; *p* <0.001), fatigue (0.71 [0.55 to 0.91]; *p* = 0.007), pain (0.72 [0.55 to 0.96]; *p* = 0.024), dyspnea (0.62 [0.45, to 0.85]; *p* = 0.003), and financial difficulties (0.60 [0.39 to 0.93]; *p* = 0.022) (Figure S1C).

**Figure 7:**
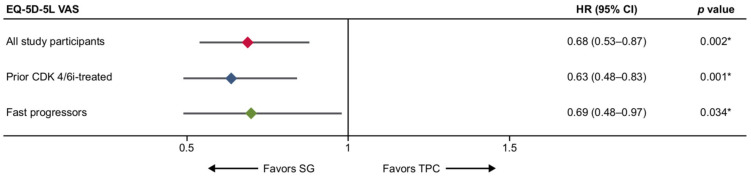
Forest plot of TTD for EQ-5D-5L VAS in overall, prior CDK4/6i-treated, and fast-progressors after not including death as an event in the analysis. * Indicate significant *p*-values (*p* < 0.05). CDK4/6i = cyclin-dependent kinase 4/6 inhibitor; CI = confidence interval; EQ-5D-5L VAS = EuroQoL Five Dimensions Five Levels Visual Analog Scale; HR = Hazard ratio; SG, sacituzumab govitecan; TPC = treatment of physician’s choice; TTD = time to deterioration.

The TTD findings remained significant for EQ-5D-5L VAS across all three populations when not including death as an event in the analysis (overall population: 0.68 [0.53 to 0.87]; *p* = 0.002, prior CDK4/6i-treated: 0.63 [0.48 to 0.83]; *p* = 0.001, fast-progressors: 0.69 [0.48 to 0.97]; *p* = 0.034) (Figure 7).

The results of the two-stage TTD analysis have been summarized in the Supplementary Material for both when not including death as an event (Figure S2) and including death as an event (Figure S3).

### 3.5. Sensitivity Analysis

The results of the sensitivity analyses after employing a one-stage model for CFB (Figures S4–S6) and a one-stage model for TTD while considering death as an event (Figures S7 and S8) are depicted in the supplement. These results were generally aligned with the base case findings, demonstrating that SG was associated with statistically significantly better PROs compared with TPC.

## 4. Discussion

To our knowledge, this is the first meta-analytic study showing humanistic outcomes associated with SG versus TPC in an HR+/HER− mBC population, which can be generalized at a global level. Efficacy data from a previous meta-analysis demonstrate that treatment with SG significantly prolonged progression-free survival and overall survival compared with single-agent chemotherapy in patients with pretreated HR+/HER− locally recurrent inoperable or mBC [27]. This study uses a similar meta-analytic framework to evaluate PRO endpoints in the pooled population derived from TROPiCS-02 and EVER-132-002 trials.

The findings of the current study demonstrated that SG significantly improved PROs compared with TPC. In the base case two-stage analysis for CFB using MMRM analysis, a significant improvement with SG versus TPC was reported on various EORTC QLQ-C30 subscales. The analysis of absolute end of treatment comparisons did not show significant improvement with SG; however, the MMRM-based CFB analysis using LSMD estimate is generally more sensitive and statistically efficient by accounting for intra-patient correlations and baseline imbalances. The results of the IPD-based meta-analysis revealed a statistically significant difference in CFB scores with SG compared with TPC on EQ-5D-5L VAS; however, the results for prior CDK4/6i-treated population and fast-progressors showed a non-significant trend toward improvement with SG. Additionally, the base case one-stage analysis demonstrated a statistically significant increase in TTD with SG over TPC on various domains of EORTC QLQ-C30 in the overall, prior CDK4/6i-treated and fast-progressors population. Sensitivity analyses using the one-stage model after including death were completely aligned with the base case one-stage results. Similarly, SG demonstrated a statistically significant extension in TTD over TPC on EQ-5D-5L VAS across all three populations.

Although PROs associated with SG were inferior to TPC for diarrhea and nausea/vomiting, superior results were observed on all other PRO domains and the QLQ-C30 summary score. These PRO benefits were observed consistently in the subgroups of patients that were previously treated with CDK4/6i and patients with fast progression. The worsening of nausea/vomiting and diarrhea associated with SG did not adversely affect GHS/QoL, QLQ-C30 summary score, or functioning and are a known part of SG’s toxicity profile. These results are consistent with published safety findings from TROPiCS-02 and EVER-132-002, where the incidence of adverse events such as nausea, diarrhea, vomiting, anemia, and neutropenia leading to treatment discontinuation were infrequent [7,28]. During the trial, adverse events including nausea/vomiting and diarrhea were managed using antiemetics, antidiarrheal agents, and other supportive measures, while grade 3/4 adverse events were managed using SG dose reductions [14]. Therefore, the evidence collectively suggests that SG has a manageable AE profile, and that management of these adverse events using established treatment guidelines can lower rates of treatment discontinuation.

The findings of our analysis were aligned with individual trial results of TROPiCS-02 and EVER-132-002, where SG showed improvement in PROs in HR+/HER− mBC. In the TROPiCS-02 trial, patients with HR+/HER− mBC receiving SG demonstrated significantly better CFB scores in physical functioning and dyspnea compared with TPC. Moreover, the time to first clinically meaningful worsening or death was significantly longer for SG on various EORTC QLQ-C30 domains including GHS/QoL, physical functioning, fatigue, emotional functioning, dyspnea, insomnia, and financial difficulties as well as the EQ-5D-5L VAS [29]. Similar findings were reported in the EVER-132-002 trial for the Asian population with HR+/HER− mBC across most of the EORTC QLQ-C30 domains [10]. Furthermore, similar results of sensitivity analyses confirmed the robustness of the base case findings, with consistent results observed across different statistical models. The ASCENT trial investigated the effect of SG versus TPC on PROs in patients with refractory/relapsed metastatic triple-negative breast cancer. The findings reported that SG demonstrated significantly better CFB scores on GHS/QoL, physical functioning, fatigue, and pain [28]. Similar findings were reported in our meta-analysis for all these outcomes, along with role functioning and dyspnea, except GHS/QoL. Similar to our study, a significant increase in time to the first clinically meaningful worsening was reported for six domains in the ASCENT trial including emotional functioning (HR 0.65 [0.52 to 0.80; *p* <0.0001), social functioning (0.69 [0.56 to 0.85]; *p* = 0.0006), insomnia (0.74 [0.59 to 0.91; *p* = 0.0054), dyspnea (0.54 [0.43 to 0.67; *p* <0.0001), financial difficulties (0.61 [0.49 to 0.77]; *p* <0.0001), and QLQ-C30 summary score (0.78 [0.63 to 0.96]; *p* = 0.0188) [30]. Collectively, the above findings highlight the reliability of the primary analysis and reinforce the evidence supporting the benefits of SG.

Our results could be contextualized by comparing PROs associated with other antibody–drug conjugates including trastuzumab deruxtecan and datopotamab deruxtecan evaluated in DESTINY-Breast04 and TROPION-Breast01, respectively; however, it is crucial to acknowledge that cross-trial comparisons should be interpreted with caution due to differences in baseline characteristics and inclusion/exclusion criteria between these trials. In the DESTINY-Breast04 trial, definitive TDD was significantly longer for the trastuzumab deruxtecan group across the EORTC QLQ-C30 domains including GHS/QoL (HR 0.71 [95% CI 0.56 to 0.92]), pain (0.51 [0.39 to 0.65]), physical functioning (0.54 [0.42 to 0.70]), and EQ-5D-5L VAS (0.70 [0.54 to 0.91]) [31]. On the other hand, the findings from the TROPION-Breast01 trial reported that patients with inoperable or HR+/HER− mBC receiving datopotamab deruxtecan had non-significantly longer TTD on various EORTC QLQ-C30 domains compared to chemotherapy with statistical significance attained in physical functioning (0.77 [0.61 to 0.99]) [32]. In our study, SG performed better than TPC in a higher number of the EORTC QLQ-C30 domains (GHS/QoL, physical functioning, emotional functioning, fatigue, pain, dyspnea, financial difficulties) and EQ-5D-5L VAS, consistent with DESTINY-Breast04 and TROPION-Breast01, where trastuzumab deruxtecan and datopotamab deruxtecan had better PROs than TPC in the respective comparator arms. Furthermore, in TTD analysis, since the definition of “event” varied across trials, a robust approach to evaluate the PRO results among SG, trastuzumab deruxtecan, and datopotamab deruxtecan warrants a feasibility assessment of indirect treatment comparison of PROs among these antibody–drug conjugates studied in TROPiCS-02/EVER-132-002, DESTINY-Breast04, and TROPION-Breast-01.

The study’s strength lies in its adherence to the PRISMA-IPD guidelines. This ensures a systematic and transparent approach to ensure consistency between reports in data synthesis, enhancing the study’s credibility and reliability [33]. Conducting a meta-analysis using IPD for time-to-event outcomes such as TTD offers more accurate estimates of treatment effects and reduces bias compared with the traditional methods [34]. IPD also effectively examines differences among patient groups and considers the impact of intercurrent events, like death, on PROs. The findings of the current study revealed that SG consistently improved TTD compared with TPC across different EORTC QLQ-C30 domains and the results remained similar even when death was included as an event. This highlights the importance of factoring in events like death when assessing PROs to ensure that conclusions about treatment benefits are accurate and reflect patients’ real experiences.

However, this study also had limitations. Although one-stage meta-analysis combines all data for increased statistical power and flexibility, it may face difficulties in addressing variations in study methodologies, populations, and other factors [35]. The differences in geography across trials, particularly the higher percentage of Asian patients in the EVER-132-002 trial, may introduce unmeasured confounders and significantly impact the PROs for patients with breast cancer [36]. Additionally, there were differences in the prior use of CDK4/6i between the trials, which could be reflective of differences in the timing of the adoption of CDK4/6i between the regions. These differences could have affected baseline characteristics, particularly the health status of the patients before treatment [34]. Patients who had received CDK4/6i might have a different history of previous lines of therapy than those who had not. Varying levels of prior exposure to various treatments could explain the observed differences in PROs between the two trials.

The timing and frequency of PRO assessments varied between the trials, particularly for CFB measures. These differences made it challenging to directly compare CFB outcomes between the trials, leading to the use of a two-stage analysis approach. Furthermore, differences in how patients perceive their health, based on cultural, psychological, or contextual factors, may introduce variability in reporting [35,36]. This subjectivity could be particularly pronounced in a global study involving patients from different countries and cultures. The analysis focused on patients with HR+/HER− mBC, which limits the generalizability of the findings to other breast cancer subtypes (e.g., triple-negative or HER2+). While SG showed improvements in PROs for this specific population, the findings may not apply to broader or more heterogeneous breast cancer populations. Lastly, since PRO questionnaires rely on patients recalling their health status over a specific time frame, there is a risk of recall bias. Patients may perceive and report their symptoms differently, particularly in long-term studies where the time between assessments can be extended.

## 5. Conclusions

In conclusion, this meta-analysis of two phase 3 randomized controlled trials demonstrated superior quality of life, as measured by PROs for SG versus TPC, confirming the results of each trial. The consistency of the results across different patient populations and several time-to-event analyses enhances the generalizability of the individual trials and reinforces the PRO benefits associated with SG versus TPC in different regional populations. These findings support the use of SG as a standard of care for pre-treated, endocrine-resistant HR+/HER− mBC irrespective of previous CDK4/6i exposure.

## Figures and Tables

**Table 1:** Participants, interventions, comparators, outcomes, and study design (PICOS) criteria for inclusion in the meta-analysis.

Criteria	TROPiCS-02	EVER-132-002
**Population**	Patients with HR+/HER2− mBC who have progressed after endocrine therapy, taxane, and at least two systemic therapies in the advanced setting	Patients with HR+/HER2− mBC who have progressed after endocrine therapy, taxane, and at least two systemic therapies in the advanced setting
	**Prior CDK4/6 inhibitor required**	**Prior CDK4/6 inhibitor not required**
**Intervention**	SG	
**Comparator**	TPC, i.e., gemcitabine, eribulin, capecitabine, vinorelbine	
**Outcome**	Patient-reported outcomes (EORTC QLQ-C30, EQ-5D-5L VAS) measured using: • Change from baseline (analyzed using MMRM; mean difference analyzed at end of treatment) • Time to deterioration (analyzed using stratified Cox proportional hazards regression analysis)	
**Prior CDK4/6 inhibitor exposure**	**Mandatory**	**Not mandatory**
**Study design**	Randomized controlled trial	

CDK4/6 = cyclin-dependent kinase 4/6; EORTC QLQ-C30 = European Organization for Research and Treatment of Cancer Quality of Life Questionnaire Version 3.0; EQ-5D-5L VAS = EuroQol 5 Dimensions 5 Levels Visual Analog Scale; HER− = human epidermal growth factor receptor 2 negative; HR+ = hormone receptor-positive; mBC = metastatic breast cancer; MMRM = mixed-effects model for repeated measures; SG = sacituzumab govitecan; TPC = treatment of physician’s choice.

**Table 2:** Study characteristics for the PRO-evaluable population.

Variable	TROPiCS-02	EVER-132-002
**Trial design**	Phase 3, randomized controlled trial	
**Blinding**	Open-label	
**Setting**	Multicenter international	
**Prior CDK4/6i treatment**	**Mandatory inclusion criterion (100% Prior CDK4/6i)**	**Not a mandatory criterion (49% Prior CDK4/6i)**
**Geography**	**Global (US, Belgium, Canada, France, Germany, Great Britain, Italy, the Netherlands, Spain)**	**Asian (China mainland, Taiwan, and Republic of Korea)**
**Stratification factors**	Prior chemotherapy regimens for treatment of metastatic disease (two versus three/four lines) and visceral metastasis (Yes/No)	Prior chemotherapy regimens for treatment of metastatic disease (two versus three/four lines) and visceral metastasis (Yes/No)
	**Endocrine therapy in metastatic setting ≥ 6 months (Yes/No)**	**Prior CDK4/6i (Yes/No)**
**ITT population**	**543 (SG: 272; TPC: 271)**	**331 (SG: 166; TPC: 165)**
**EORTC QLQ-C30-evaluable population**	**446 (SG: 236; TPC: 210)**	**318 (SG: 161; TPC: 157)**
**EQ-5D-5L-Evaluable Population**	**445 (SG: 238; TPC: 207)**	**318 (SG: 161; TPC: 157)**
**Data-cut off**	**1 December 2022**	**30 April 2023**
**Treatment**	SG 10 mg/kg IV on Days 1 and 8 of 21-day cycles	
**Comparator**	TPC (a single-agent treatment determined by the investigator before randomization from one of the four following choices: eribulin, capecitabine, gemcitabine, or vinorelbine)	
**Treatment duration, median (SG versus TPC)**	**4.1 months vs. 2.3 months**	**5.1 months versus 3.25 months**
**Follow-up time**	**PRO questionnaires evaluated in all patients at baseline (within 3 days of first study treatment), Day 1 of every cycle except Cycle 1 (every 3 weeks for TPC if given weekly), and the final study visit (prior to telling patients that they are being withdrawn from the study); median duration: 12.75 months**	**PRO assessments evaluated on C1D1, at each tumor assessment visit, and the end of treatment visit. The assessment of PROs was before tumor assessments at the planned visit; median duration: 13.4 months**
**Measures being evaluated**	Patient-reported outcomes (EORTC QLQ-C30, EQ-5D-5L VAS) measured using: • Change from baseline • Time to deterioration	

CDK4/6i = cyclin-dependent kinase 4/6 inhibitor; C1D1 = Cycle 1 Day 1; EORTC QLQ-C30 = European Organization for Research and Treatment of Cancer Quality of Life Questionnaire Version 3.0; EQ-5D-5L = EuroQol 5 Dimensions 5 Levels; IV = intravenous; PROs = patient-reported outcomes; SG = sacituzumab govitecan; TPC = treatment of physician’s choice; US = United States; VAS = Visual analog scale. In TROPiCS-02, participants were from the US (42.0%), France (25.2%), Spain (12.7%), Germany (8.5%), Belgium (4.6%), Italy (2.8%), Great Britain (2.6%), the Netherlands (1.5%), Canada (0.2%). In EVER-132-002, participants were from the geographical regions of China (70.1%), Republic of Korea (21.1%), and Taiwan (8.8%).

## Data Availability

Additional data not included in the supplement are available on request from the corresponding author.

## Supplementary Materials

The following supporting information can be downloaded at: https://www.mdpi.com/article/10.3390/cancers17111885/s1, Figure S1. Forest plot of TTD in the various scales of the EORTC QLQ-C30 questionnaire in overall (A), prior CDK4/6i-treated (B), and fast-progressors (C) after not including death as an event in the analysis. * Indicate significant *p*-values (*p* < 0.05). Figure S2. Two-stage results of TTD for EORTC QLQ-C30 and EQ-5D-5L VAS death as censored in overall, prior CDK4/6i-treated and fast-progressors population. * Indicate significant *p*-values (*p* < 0.05). Figure S3. Two-stage results of TTD for EORTC QLQ-C30 and EQ-5D-5L VAS death as event in overall, prior CDK4/6i-treated and fast-progressors population. * Indicate significant *p*-values (*p* < 0.05). Figure S4. One-stage results for treatment comparison of mean CFB in functional domains, GHS, summary scores, financial difficulties of EORTC QLQ-C30 of overall, prior CDK4/6i-treated and fast-progressors population. * Indicate significant *p*-values (*p* < 0.05). Figure S5. One-stage results for treatment comparison of mean CFB in symptom domain scores of EORTC QLQ-C30 in overall, prior CDK4/6i-treated and fast-progressors population. * Indicate significant *p*-values (*p* < 0.05). Figure S6. One-stage results for treatment comparison of mean CFB EQ-5D-5L VAS in overall, prior CDK4/6i-treated and fast-progressors population. * Indicate significant *p*-values (*p* < 0.05). Figure S7. One-stage TTD results for various scales of the EORTC QLQ-C30 questionnaire in overall (A), prior CDK4/6i-treated (B), and fast-progressors (C) in death as event analysis. * Indicate significant *p*-values (*p* < 0.05). Figure S8. One-stage TTD results for EQ-5D-5L VAS in overall, prior CDK4/6i-treated, and fast-progressors in death as event analysis. * Indicate significant *p*-values (*p* < 0.05). Table S1. Summary of baseline characteristics of studies.
