# Supplementary material for: A Meta-Analysis of Patient-Reported Outcomes of Sacituzumab Govitecan Versus Treatment of Physician’s Choice in Previously Treated HR+/HER− mBC Using Two Phase 3 (TROPiCS-02 and EVER-132-002) Trials
Source: Cancers (Basel). 2025 Jun 4;17(11):1885. doi: 10.3390/cancers17111885 (PMC12153749; doi:10.3390/cancers17111885)
Supplement: Supplementary file 1 [file cancers-17-01885-s001.zip › cancers-3565021-supplementary.pdf]

Supplementary Material

Table S1. Summary of baseline characteristics of studies.

Variable	TROPiCS-02 (N = 543)	EVER-132-002 (N = 331)
**Overall population (Irrespective of CDK4/6i treatment status)**		
Age (%)	<65 years: 74.2<br>≥65 years: 25.8	<65 years: 88.5<br>≥65 years: 11.5
Mean age	56.4	52.1
Females (%)	99.1	99.4
Race (%)	American Indian or Alaska Native: 0<br>Asian: 2.9<br>Black or African American: 3.9<br>Multiple: 0.2<br>Native Hawaiian or Other Pacific Islander: 0.2<br>White: 66.7<br>Other: 0.6<br>Not reported: 25.6	American Indian or Alaska Native: 0<br>Asian: 100<br>Black or African American: 0<br>Multiple: 0<br>Native Hawaiian or Other Pacific Islander: 0<br>White: 0<br>Other: 0<br>Not reported: 0
Ethnicity (%)	Hispanic or Latino: 3.3<br>Not Hispanic or Latino: 78.5<br>Unknown: 5.2<br>Not Reported: 11.2<br>Missing: 1.8	Hispanic or Latino: 0<br>Not Hispanic or Latino: 0<br>Unknown: 0<br>Not Reported: 100<br>Missing: 0
Region (%)	North America: 42.2<br>Europe: 57.8<br>Asia: --	North America: --<br>Europe: --<br>Asia: 100
ECOG PS (%)	0: 44.4<br>1: 55.6	0: 22.4<br>1: 77.6
Baseline target/nontarget liver lesion per RECIST v1.1 (%)	Yes: 85.8<br>No: 14.2	Yes: 70.7<br>No: 29.3
Time from metastatic disease to randomization	Mean 54.4 months	Mean 43.6 months
Visceral disease (%)	Yes: 95.2<br>No: 4.8	Yes: 88.5<br>No: 11.5
Prior systemic anticancer regimens	Mean 7.4	Mean 6.0
Prior lines of chemotherapy in the metastatic setting (%)	>1: 98.0<br>1: 1.8	>1: 94.6<br>1: 5.4
Prior CDK4/6i use (%)	≤12 months: 60.2<br>>12 months: 38.3<br>Missing: 1.5	≤12 months: 32.9<br>>12 months: 15.7<br>Missing: 51.4
Pre-selected TPC choice (%)	Capecitabine: 8.1<br>Eribulin: 47.5<br>Vinorelbine: 21.4<br>Gemcitabine: 23.0	Capecitabine: 5.7<br>Eribulin: 81.9<br>Vinorelbine: 7.6<br>Gemcitabine: 4.8
Treatment distribution (%)	Capecitabine: 4.1<br>Eribulin: 23.9<br>Vinorelbine: 11.6<br>Gemcitabine: 10.3<br>SG: 50.1	Capecitabine: 3.3<br>Eribulin: 39.6<br>Vinorelbine: 3.9<br>Gemcitabine: 3<br>SG: 50.2
Number of prior systemic anticancer	1: 0.2	1: --

Variable	TROPiCS-02 (N = 543)	EVER-132-002 (N = 331)
regimens by category (%)	2: 13.6<br>3: 31.7<br>4: 33.3<br>5: 16.4<br>6: 4.1<br>7: 0.6<br>9: 0.2	2: 18.4<br>3: 39.6<br>4: 30.5<br>5: 10.9<br>6: 0.3<br>7: 0.3
Prior endocrine therapy in the metastatic setting for at least 6 months (%)	Yes: 86.4<br>No: 13.6	Yes: 77.6<br>No: 22.4
Early relapse (%)	Yes: 7.7<br>No: 89.9<br>Unknown: 2.4	Yes: 10.0<br>No: 89.4<br>Unknown: 0.6
Chemotherapy in neo/adjuvant setting (%)	Yes: 65.7<br>No: 34.3	Yes: 71.9<br>No: 28.1
Prior anthracycline use (%)	Yes: 79.7<br>No: 20.3	Yes: 83.7<br>No: 16.3
UGT1A1 Genotype (in SG arm only) (%)	*1|*1: 38.2<br>*1|*6: --<br>*27|*28: --<br>*6|*28: --<br>*6|*6: --<br>*1|*28: 43.8<br>*1|*37: --<br>*28|*28: 9.2<br>*28|*36: --<br>Missing: 7.7	*1|*1: 46.6<br>*1|*6: 24.1<br>*27|*28: 1.2<br>*6|*28: 1.8<br>*6|*6: 3.0<br>*1|*28: 10.8<br>*1|*37: --<br>*28|*28: 1.8<br>*28|*36: --<br>Missing: 10.2
TROP-2 expression, H-score (%)	100 ≤ Trop-2 H-Score ≤200: 34.1<br>Trop-2 H-Score <100: 35.4<br>Trop-2 H-Score >200: 15.7	100 ≤ Trop-2 H-Score ≤200: --<br>Trop-2 H-Score <100: --<br>Trop-2 H-Score >200: --
HER2 IHC result (%)	HER2 IHC-0: 37.1<br>HER2-Low: 54.8	HER2 IHC-0: 29.9<br>HER2-Low: 70.1

CDK4/6i = cyclin-dependent kinase 4/6 inhibitor; ECOG PS = Eastern Cooperative Oncology Group Performance status; HER2 = human epidermal growth factor receptor 2; IHC = immunohistochemistry; N = sample size; RECIST = Response Evaluation Criteria in Solid Tumors; SG = sacituzumab govitecan; TPC = treatment of physician's choice; TROP-2 = trophoblast cell surface antigen-2.

In TROPiCS-02, participants were from the United States (42.0%), France (25.2%), Spain (12.7%), Germany (8.5%), Belgium (4.6%), Italy (2.8%), Great Britain (2.6%), Netherlands (1.5%), Canada (0.2%).

In EVER-132-002, participants were from the geographical regions of China (70.1%), Republic of Korea (21.1%), and Taiwan (8.8%).

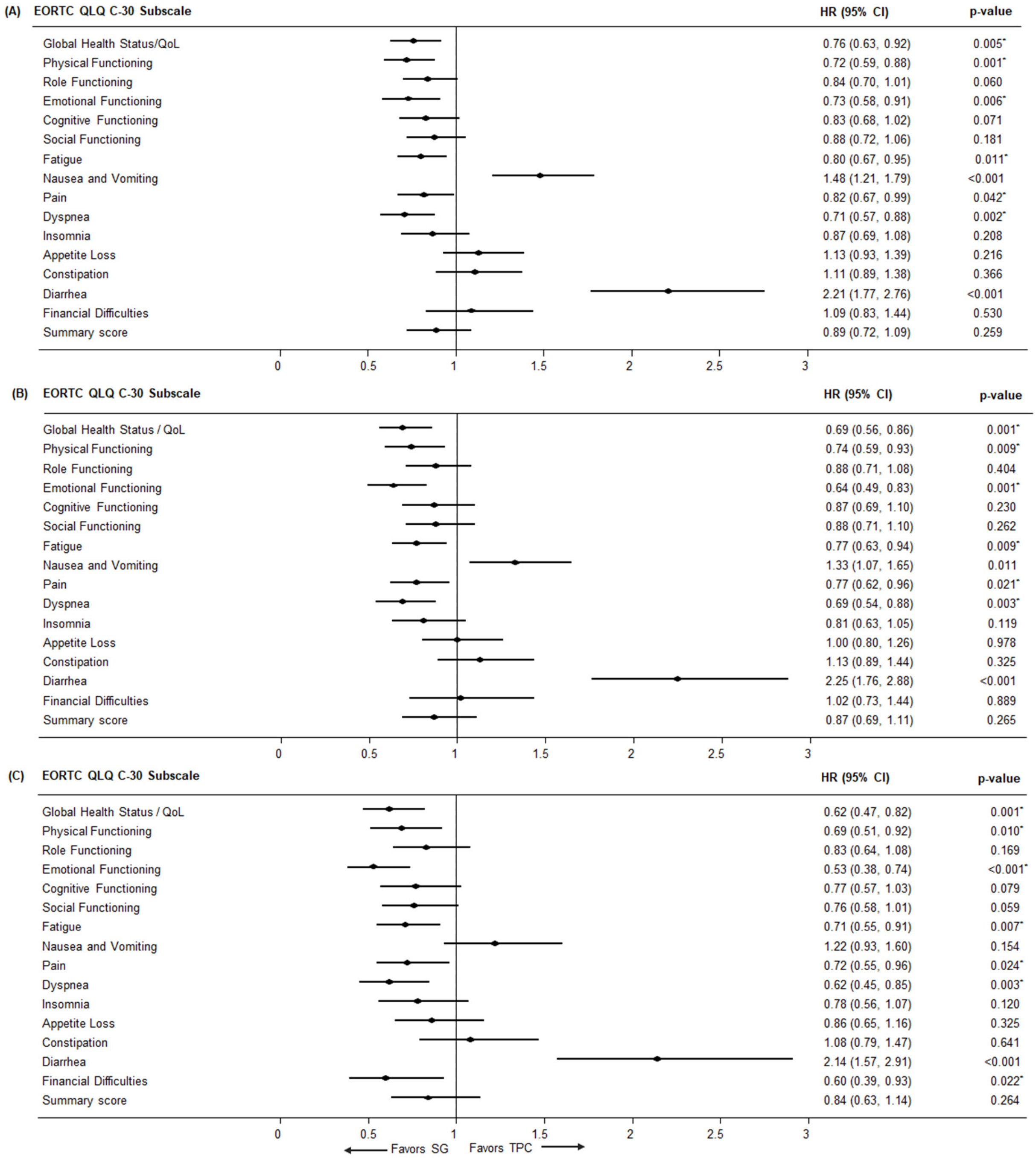

Figure S1. Forest plot of TTD in the various scales of the EORTC QLQ-C30 questionnaire in overall (A), prior CDK4/6i-treated (B), and fast-progressors (C) after not including death as an event in the analysis. * Indicate significant *p*-values (p <0.05). CDK4/6i = cyclin-dependent kinase 4/6 inhibitor; CI = confidence interval; EORTC QLQ-C30 = European Organization for Research and Treatment of Cancer Quality of Life Questionnaire Version 3.0; HR = hazard ratio; QoL = quality of life; SG = sacituzumab govitecan; TPC = treatment of physician's choice; TTD, time to deterioration.

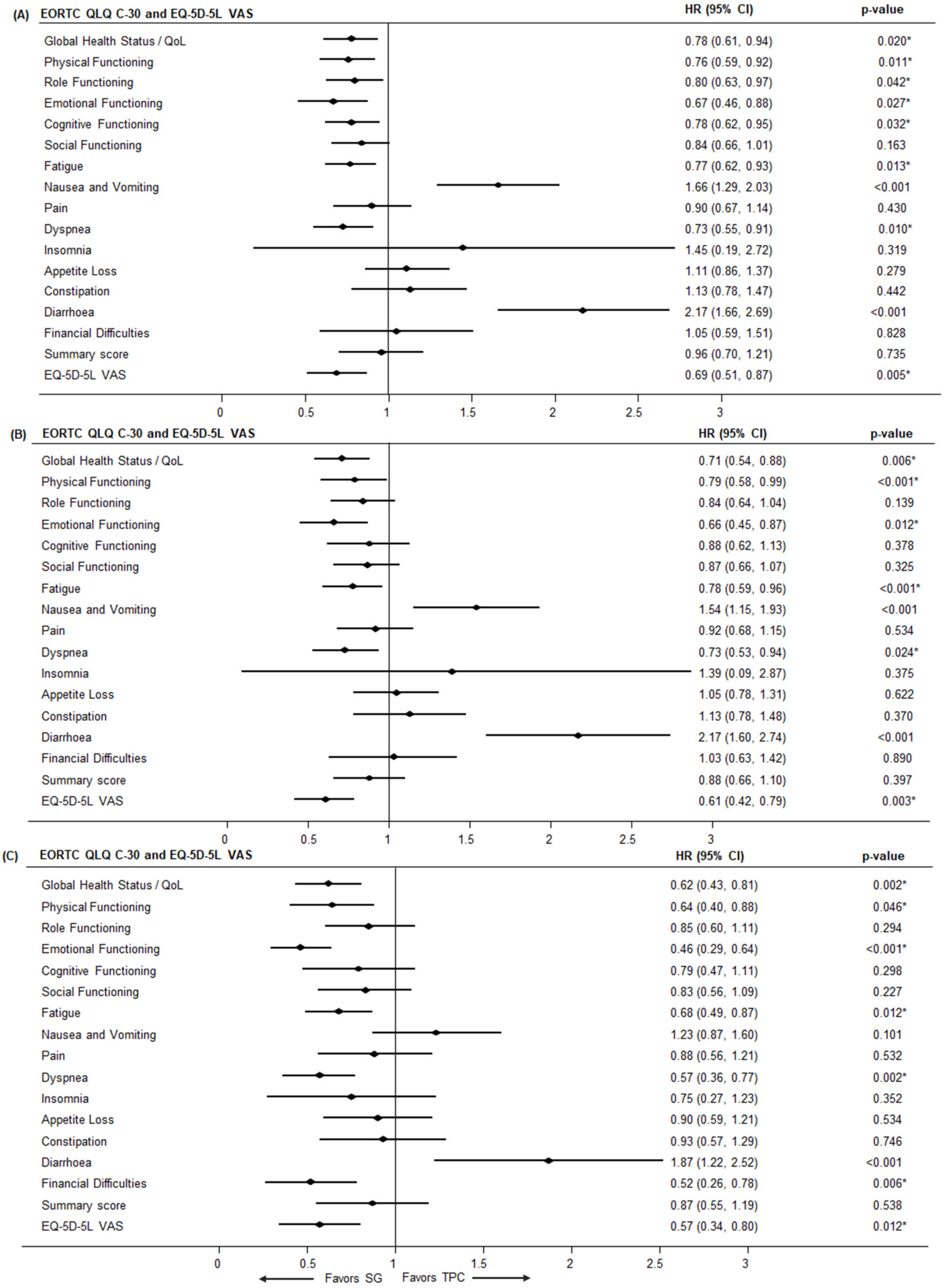

Figure S2. Two-stage results of TTD for EORTC QLQ-C30 and EQ-5D-5L VAS death as censored in overall, prior CDK4/6i-treated and fast-progressors population. * Indicate significant *p*-values ($p < 0.05$). CI = confidence interval; EORTC QLQ-C30 =, European Organization for Research and Treatment of Cancer Quality of Life Questionnaire Version 3.0; EQ-5D-5L VAS = EuroQoL Five Dimensions Five Levels Visual Analog Scale; HR = hazard ratio; QoL = quality of life; SG = sacituzumab govitecan; TPC = treatment of physician's choice; TTD, time to deterioration.

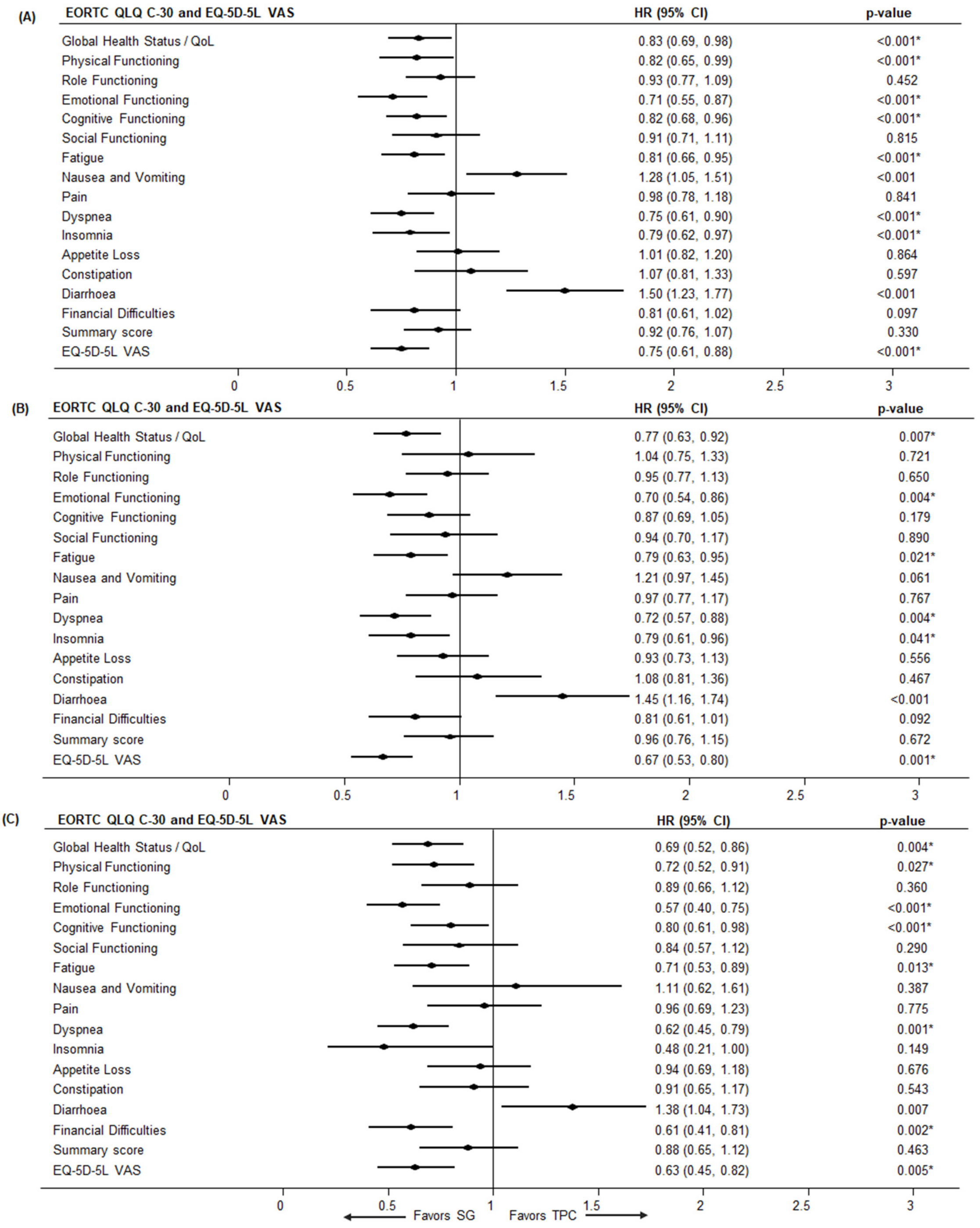

Figure S3. Two-stage results of TTD for EORTC QLQ-C30 and EQ-5D-5L VAS death as event in overall, prior CDK4/6i-treated and fast-progressors population. *Indicate significant *p*-values (p <0.05).
CDK4/6i = cyclin-dependent kinase 4/6 inhibitors; CI = Confidence interval; EORTC QLQ-C30, European Organization for Research and Treatment of Cancer Quality of Life Questionnaire Version 3.0; EQ-5D-5L VAS, EuroQoL Five Dimensions Five Levels Visual Analog Scale; HR, Hazard ratio; QoL, Quality of life; SG, Sacituzumab govitecan; TPC, Treatment of physician's choice; TTD, Time to deterioration.

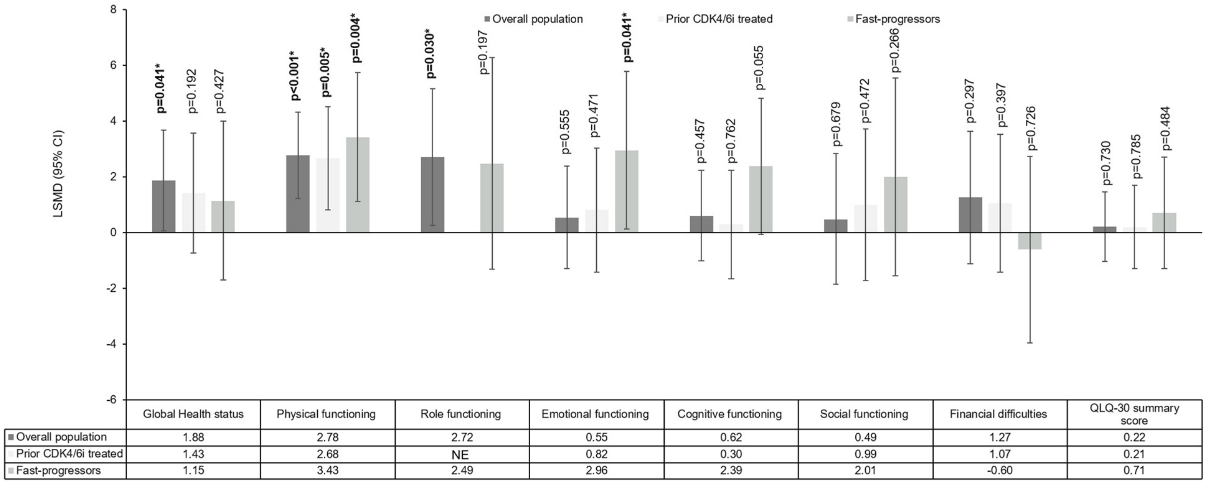

	Global Health status	Physical functioning	Role functioning	Emotional functioning	Cognitive functioning	Social functioning	Financial difficulties	QLQ-30 summary score
Overall population	1.88	2.78	2.72	0.55	0.62	0.49	1.27	0.22
Prior CDK4/6i treated	1.43	2.68	NE	0.82	0.30	0.99	1.07	0.21
Fast-progressors	1.15	3.43	2.49	2.96	2.39	2.01	-0.60	0.71

Figure S4. One-stage results for treatment comparison of mean CFB in functional domains, GHS, summary scores, financial difficulties of EORTC QLQ-C30 of overall, prior CDK4/6i-treated and fast-progressors population. *Indicate significant *p*-values ($p < 0.05$).

CDK4/6i, Cyclin-dependent kinase 4/6 inhibitor; CFB, Change from baseline; CI, Confidence interval; EORTC QLQ-C30, European Organization for Research and Treatment of Cancer Quality of Life Questionnaire Version 3.0; GHS, Global health status; LSMD, Least square mean difference; NE, Non-estimable

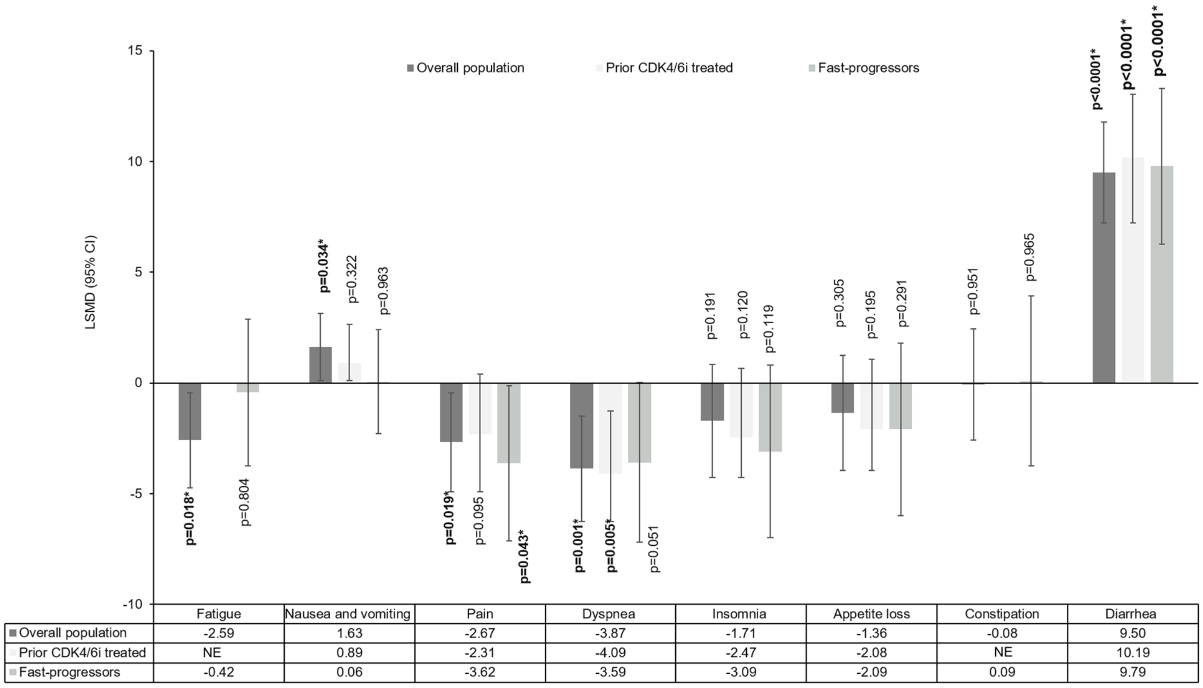

	Fatigue	Nausea and vomiting	Pain	Dyspnea	Insomnia	Appetite loss	Constipation	Diarrhea
Overall population	-2.59	1.63	-2.67	-3.87	-1.71	-1.36	-0.08	9.50
Prior CDK4/6i treated	NE	0.89	-2.31	-4.09	-2.47	-2.08	NE	10.19
Fast-progressors	-0.42	0.06	-3.62	-3.59	-3.09	-2.09	0.09	9.79

Figure S5. One-stage results for treatment comparison of mean CFB in symptom domain scores of EORTC QLQ-C30 in overall, prior CDK4/6i-treated and fast-progressors population. *Indicate significant *p*-values ($p < 0.05$).

CDK4/6i, Cyclin-dependent kinase 4/6 inhibitor; CFB, Change from baseline; CI, Confidence interval; EORTC QLQ-C30, European Organization for Research and Treatment of Cancer Quality of Life Questionnaire Version 3.0; LSMD, Least square mean difference; NE, Non-estimable

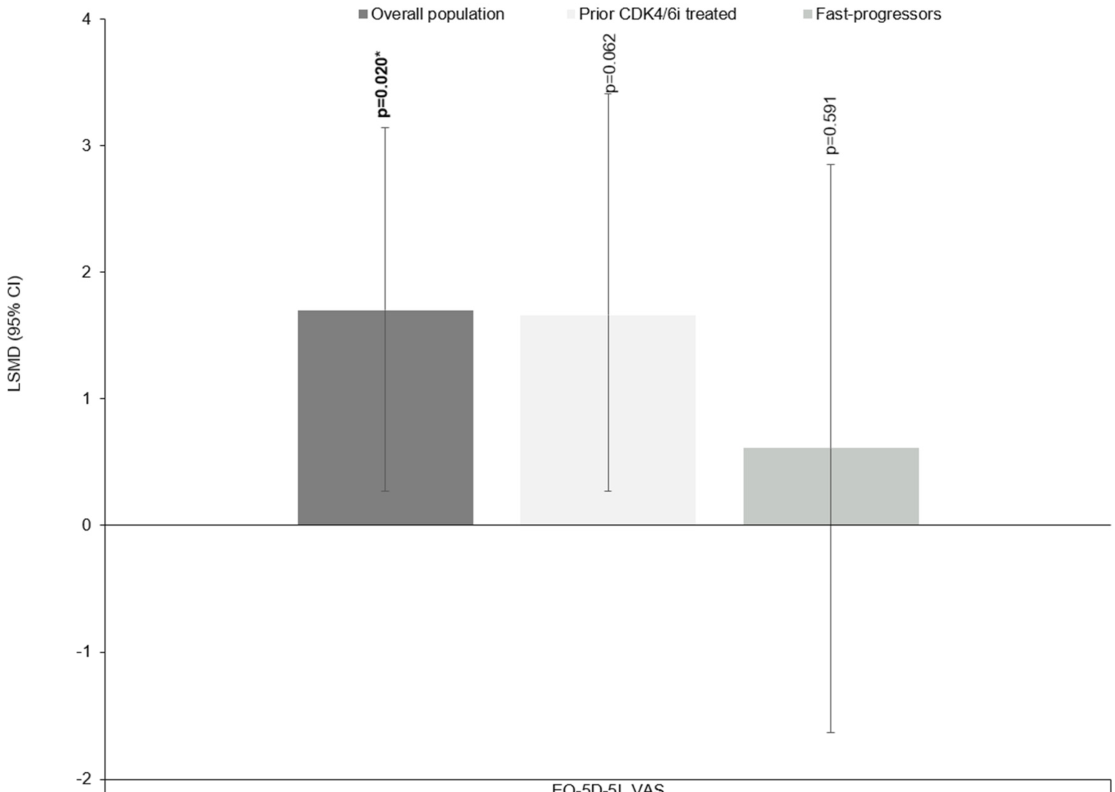

	EQ-5D-5L VAS
Overall population	1.70
Prior CDK4/6i treated	1.66
Fast-progressors	0.61

Figure S6. One-stage results for treatment comparison of mean CFB EQ-5D-5L VAS in overall, prior CDK4/6i-treated and fast-progressors population. *Indicate significant *p*-values (p <0.05).

CDK4/6i, Cyclin-dependent kinase 4/6 inhibitor; CFB, Change from baseline; CI, Confidence interval; EQ-5D-5L VAS, EuroQoL Five Dimensions Five Levels Visual Analog Scale; LSMD, Least square mean difference

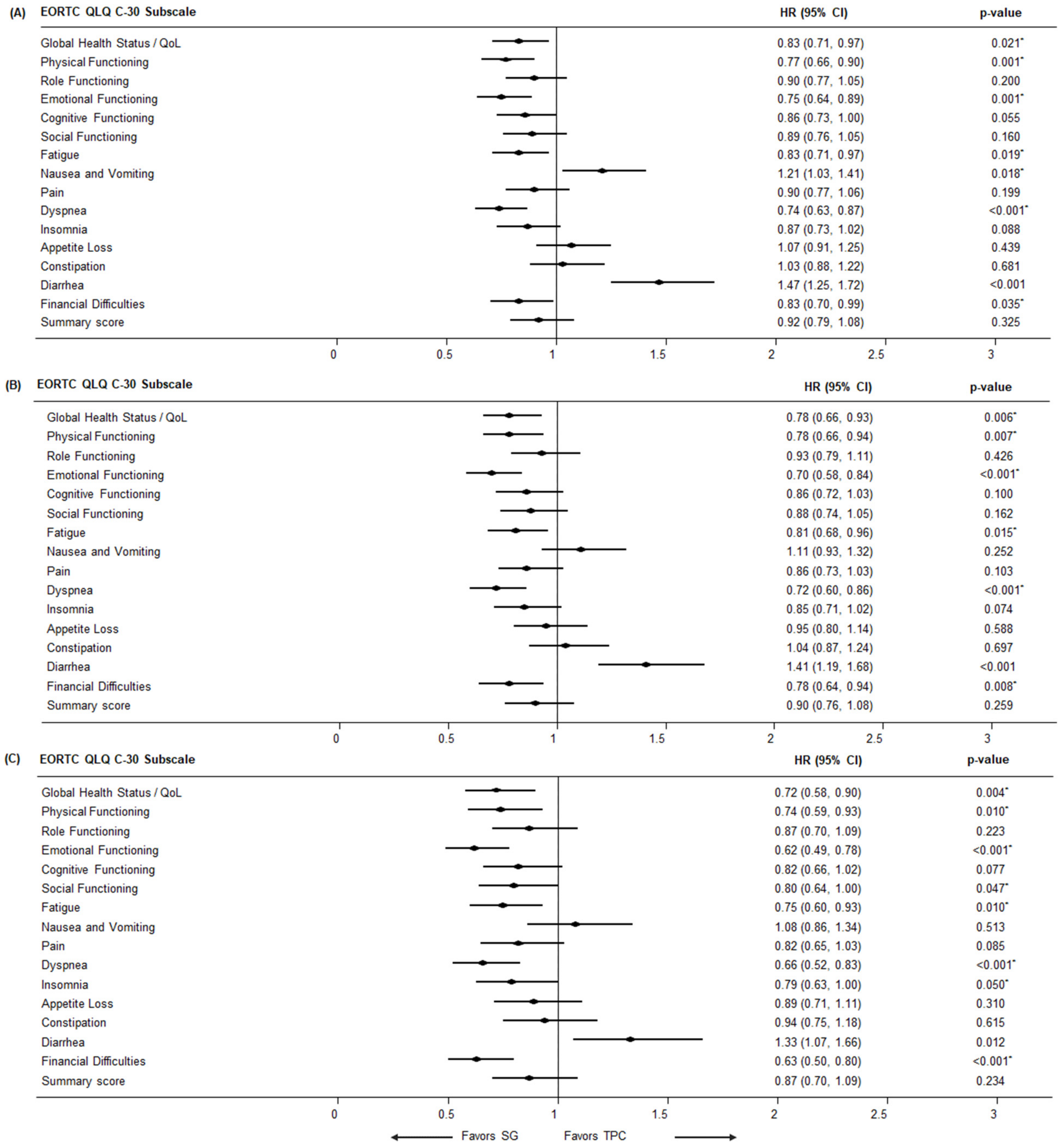

Figure S7. One-stage TTD results for various scales of the EORTC QLQ-C30 questionnaire in overall (A), prior CDK4/6i-treated (B), and fast-progressors (C) in death as event analysis. *Indicate significant *p*-values (p <0.05).

CDK4/6i, Cyclin-dependent kinase 4/6 inhibitor; CI, Confidence interval; EORTC QLQ-C30, European Organization for Research and Treatment of Cancer Quality of Life Questionnaire Version 3.0; HR, Hazard ratio; SG, Sacituzumab govitecan; TPC, Treatment of physician's choice; TTD, Time to deterioration; QoL, Quality of life

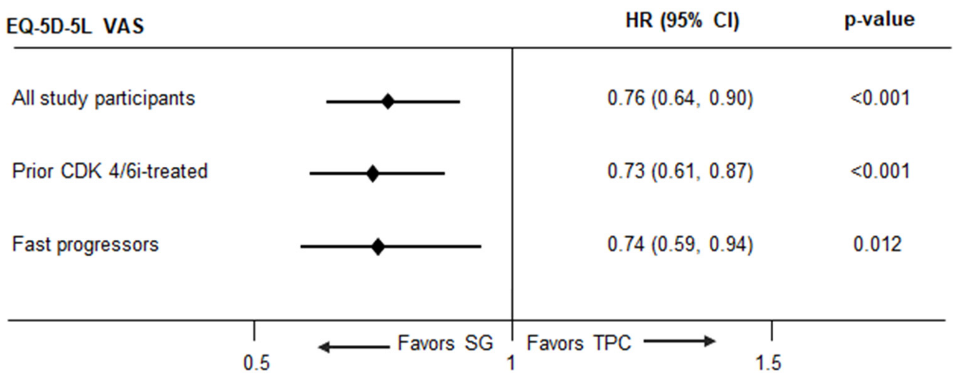

Figure S8. One-stage TTD results for EQ-5D-5L VAS in overall, prior CDK4/6i-treated, and fast-progressors in death as event analysis. *Indicate significant *p*-values (p <0.05).
CDK4/6i, Cyclin-dependent kinase 4/6 inhibitor; CI, Confidence interval; EQ-5D-5L VAS, EuroQoL Five Dimensions Five Levels Visual Analog Scale; HR, Hazard ratio; SG, Sacituzumab govitecan; TPC, Treatment of physician's choice; TTD, Time to deterioration
